# Titanium Nitride Thin Film Based Low-Redox-Interference Potentiometric pH Sensing Electrodes

**DOI:** 10.3390/s21010042

**Published:** 2020-12-23

**Authors:** Shimrith Paul Shylendra, Wade Lonsdale, Magdalena Wajrak, Mohammad Nur-E-Alam, Kamal Alameh

**Affiliations:** 1 Electron Science Research Institute, School of Science, Edith Cowan University, Joondalup 6027, Australia; m.nur-e-alam@ecu.edu.au (M.N.-E.-A.); k.alameh@ecu.edu.au (K.A.); 2 School of Science, Edith Cowan University, Joondalup 6027, Australia; w.lonsdale@ecu.edu.au (W.L.); m.wajrak@ecu.edu.au (M.W.)

**Keywords:** titanium nitride (TiN), potentiometric pH sensor, radio frequency magnetron sputtering (RFMS), redox interference

## Abstract

In this work, a solid-state potentiometric pH sensor is designed by incorporating a thin film of Radio Frequency Magnetron Sputtered (RFMS) Titanium Nitride (TiN) working electrode and a commercial Ag|AgCl|KCl double junction reference electrode. The sensor shows a linear pH slope of −59.1 mV/pH, R^2^ = 0.9997, a hysteresis as low as 1.2 mV, and drift below 3.9 mV/h. In addition, the redox interference performance of TiN electrodes is compared with that of Iridium Oxide (IrO_2_) counterparts. Experimental results show −32 mV potential shift (E^0^ value) in 1 mM ascorbic acid (reducing agent) for TiN electrodes, and this is significantly lower than the −114 mV potential shift of IrO_2_ electrodes with sub-Nernstian sensitivity. These results are most encouraging and pave the way towards the development of miniaturized, cost-effective, and robust pH sensors for difficult matrices, such as wine and fresh orange juice.

## 1. Introduction

The pH scale is well-known, where colloquially, a pH of 7 is neutral, whilst acidity increases as the number lowers and alkalinity increases as the number gets higher. The concept of pH was first introduced back in 1909, and shortly after pH was defined as the negative logarithm of the hydrogen-ion activity in solution [1,2] as shown in Equation (1).

pH = −log α[H^+^] (1)

Among the measured parameters, the pH value is the most frequently measured [3,4]. For example, in agriculture, the pH of soil affects crop yield [5]; in nature, the pH of sea water plays a vital role in the ocean’s carbon cycle [6]; in the brewing industry, pH is important during the brewing process [7,8,9]; in microbiology [10], sweat monitoring [11]. Intercellular pH is maintained at neutrality as this is the pH at which the charge in the cell gets trapped inside [12,13,14]. In manufacturing industries, like portable water purification plants, the pH of drinking water is set forth by regulatory agencies; hence, it must be within the specified range [15]. There are numerous applications, which require reliable pH sensors [16].

The glass pH electrode is the most reliable option for pH measurements. Glass pH electrodes consist of a double junction single shaft combining a working electrode and reference electrode (Ag|AgCl) in contact with an electrolyte (KCl, 3 mol/L). The potential difference that occurs across the glass membrane depends on the H^+^ activity [17]. The pH of the solution is determined using the Nernst Equation [18]. Glass electrodes of different sensitivities have been used for the development of specific pH sensors for various sensing applications. While glass electrodes have several advantages, such as, Nernstian sensitivity, superior ion selectivity, excellent stability, and extensive operating range, they have major disadvantages, including the requirement for storage in wet condition, fragility, manufacturing cost, and limited size and shape, which make them impractical for applications like an endoscopic sensor for biomedical application, and soil pH testing in a harsh environment [19,20,21,22]. Glass electrodes are inapplicable in micro profiles in a wide range of microenvironments, which require portability and viability. To overcome these issues of glass electrodes, various metal oxides have been investigated and proposed as potential electrode materials for the development of pH sensors, as they offer unique features, such as, insolubility, stability, mechanical strength, and the possibility of miniaturization [4]. However, the main drawback of metal-oxide pH sensors is the interference caused by oxidizing and reducing agents in the sample solution. Most metal oxides are electrically conductive. Nevertheless, they are also sensitive to redox species, e.g., ferricyanide ion in the sample, which influences the pH measurements and results in significant errors [4] and making them impractical for the detection of the trace levels of analytes.

In order to reduce such kind of error, metal nitride is considered as an alternative sensing material. Compared to other chemical sensor materials, metal nitrides exhibit higher chemical inertness, better wear, and high electron mobility [23]. Additionally, nitrides have a greater energy bandgap, high melting point, increased hardness, brittleness, and excellent thermal and electrical conductivity [23]. Nitrogen atoms in the metal nitrides occupy interstitial sites in the metal and are believed to promote strong metal-to-non-metal and metal-to-metal bonds [23]. Specifically, in applications, which require electronic devices, field emission, and electrochemical capacitors, TiN has been proven as a suitable conducting membrane. Therefore, in this study, metal nitrides thin films were investigated as an alternative to metal oxides for the development of pH sensors. The pH sensing properties of metal nitrides, along with the effects of redox agents on metal nitrides, will particularly be investigated for the development of potentiometric pH sensors. To date, metal nitrides have mainly been used for Ion Selective Field Effect Transistor (ISFET) type pH sensors RuN, *α-BC_x_N_y_*, TiN, Si_3_N_4_ [20,24,25,26,27]. For ruthenium nitride (RuN), the pH sensing and non-ideal characteristics of the sensing membrane have been investigated and reported by Y.H. Liao et al. [24]. The I_D_- V_G_ curves determine the sensitivity of the sensing membrane by a current-voltage(I-V) system in buffer solutions. According to Y.H. Liao et al. [24], in a (Metal Oxide Semiconductor Field effect Transistor) MOSFET-based pH sensing membrane, the gate of MOSFET is connected to the RuN pH sensor and drain, the source is connected to the current unit. Both pH sensor device and an Ag/AgCl reference electrode are immersed in a standard buffer solution, while a constant potential is maintained throughout the measurement. The drain-source voltage (V_DS_) is kept constant, and V_G_ voltage is increased while the drain-source current (I_DS_) is measured. I_D_ –V_G_ curves determines the sensitivity of RuN sensing membrane. The sensing characteristics of the ruthenium nitride membrane were stable in pH buffer solution 1 to 13, sensitivity of the RuN sensor was 58.03 mV/pH. Although, these devices have high pH sensitivity, ISFET based sensors show some disadvantages including long-term drift, hysteresis, and thermal drift, which limits the accuracy of the pH measurement. Previous work carried out in 1997 on metal nitrides showed that, for example, zirconium nitride (ZrN) and hafnium nitride (HfN), have exceptional surface-stability because of their interlinked mixture of covalent and ionic bonds [28]. Subsequently, research work on metal nitrides was conducted in 2001, where, a pH sensor integrating Complementary Metal Oxide Semiconductor (CMOS) in conjunction with an Extended Gate Field Effect Transistor (EGFET) was fabricated on the same chip using titanium nitride (TiN) as a sensing membrane, demonstrating a sensitivity of −57 mV/pH [27,29]. Later, in 2011, Y.H Chang et al. [23] showed that Group-III nitrides, including GaN, AlN, InN, and their alloys are promising materials for next-generation chemical and biological sensors. Finally, in 2013, K. A. Yusof et al. [26] developed and investigated the performances of a Si_3_N_4_ based ISFET sensor and a Si_3_N_4_ as a thin film sensor and demonstrating sensitivities of −53.5 mV/pH and −66.9 mV/pH, respectively.

Despite the work that has been reported on the use of metal nitrides as a pH sensing material, there are still several gaps in this field of research that need to be addressed for developing future-generation ultrafast and miniaturized pH sensors. Firstly, metal nitrides have mainly been used for developing ISFET-based pH sensors, not potentiometric sensors. A thorough investigation of the potentiometric pH sensing mechanism of metal nitrides to detect the pH level is lacking in the literature. Secondly, the only metal nitride reported for potentiometric pH sensing was TiN [20]. However, no study on the redox matrix was conducted. TiN is an electrically conducting material with good mechanical properties, high corrosion resistance, and biocompatibility, which makes it a promising material for novel pH sensors.

In this work, titanium nitride as a pH sensing material has been proposed as an alternative to noble metals, such as gold, platinum, and iridium, and for existing pH sensing materials. A comprehensive study of TiN thin films fabrication and characterization is presented here, which provides a better understanding of the behavior of TiN as a potential pH sensing layer and its redox effects. TiN layers are manufactured by using RF magnetron sputtering. The effect of sputtering process parameters, such as layer thickness, RF power density to the target material, and gas composition are investigated to optimize the sputtering process parameters for obtaining the best materials properties suitable for using this film as a pH sensor paired up with a commercial Ag|AgCl|KCl double junction reference electrode. In addition, the underlining mechanism that governs the pH sensitivity of titanium nitrides is investigated by experimentally testing the pH sensing properties of TiN films (i.e., sensitivity, hysteresis, and drift) for potentiometric sensing. The effect of redox agents on the TiN sensing material has also been experimentally tested and compared with IrO_2_-based sensing materials in order to broaden TiN sensor applications. Additionally, the unprotected TiN film is tested under high redox matrices; white wine and fresh orange juice.

## 2. Materials and Methods

### 2.1. Working Electrodes Fabrication

Titanium nitride pH working-electrodes were prepared using an RF Magnetron sputtering system on various substrates such as alumina (Al_2_O_3_), glass, silicon (Si), and polyimide (PI) (plastic). Sputtered-deposition process parameters used for fabrication of metal-nitride thin-films under a controlled process to produce the solid-state pH sensors with excellent performance are summarized in Table 1 below.

**Figure 1:**
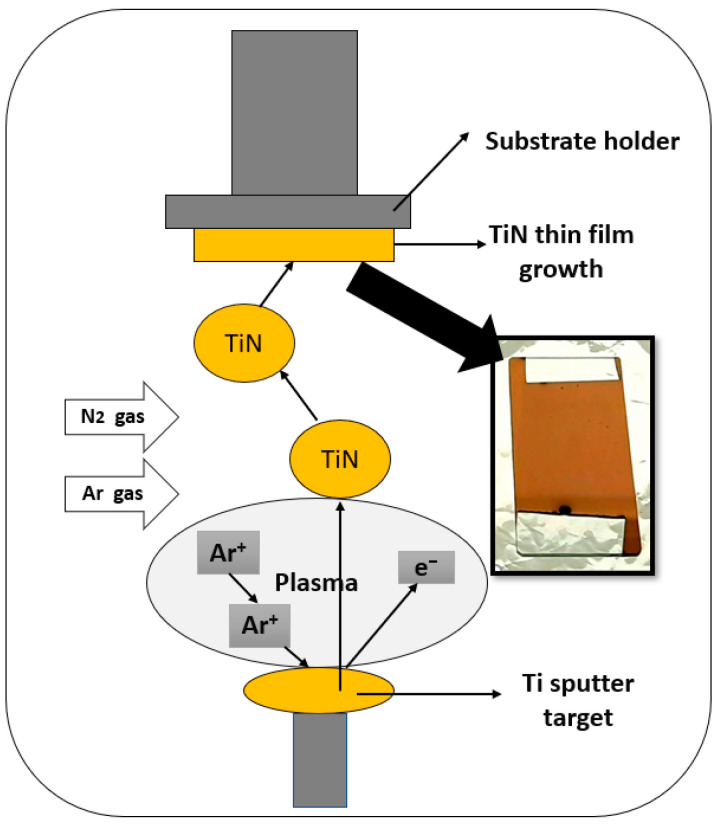
Schematic diagram of radio frequency (RF) Magnetron sputtering system used to prepare the TiN thin-film layers. Insert showing the image of the deposited TiN film on the glass substrate.

The substrates were cleaned using acetone and isopropyl alcohol in an ultrasonic bath for 15–30 min, after which they were completely dried on a hot plate at 120 °C. A titanium nitride layer of a thickness of 85 nm was sputtered from a 4-inch diameter titanium target (99.95% purity). Figure 1 shows a schematic diagram of an RF magnetron sputtering system where a Titanium (Ti) metallic target of 4-inch (10.16 cm) diameter has been installed inside the chamber. TiN thin films about 85 nm were deposited by allowing nitrogen during the deposition process. The gold-colored titanium nitride coated films on glass substrate confirmed the presence of TiN layer on the substrates, as can be seen in the insert of Figure 1.

### 2.2. pH Characteristics by Potentiometric Method

An Atlas Scientific ORP EZO circuit was used to record potential differences between the pH electrodes (reference and working electrodes) in real-time, connected to a PC via an electrically isolated USB EZO carrier board, as a potentiometric setup. The working electrodes were cleaned with blasts of air at each measurement. The electrode was equilibrated in pH 7 overnight before the actual test to obtain stable potential readings. The potential was recorded with a 70 s interval in Rowe Scientific commercial pH 2, pH 4, pH 7, pH 10, and pH 12 buffer solutions at 22 °C, coupled with a commercial Ag|AgCl|KCl double junction glass reference electrode. The pH acidic and alkaline loop cycles of 7−4−7−10−7 and 7−2−7−12−7 were used for testing and repeated 3 times, of which the last 30 s potential values were averaged to calculate the sensitivity, E^0^, and hysteresis, which represented the difference of consecutive measurements at pH 7, and drift, which represented the shift in potential at pH 7 over the entire test period.

## 3. Results and Discussion

### 3.1. Characteristics Results of Sputtered TiN Films

**Figure 2:**
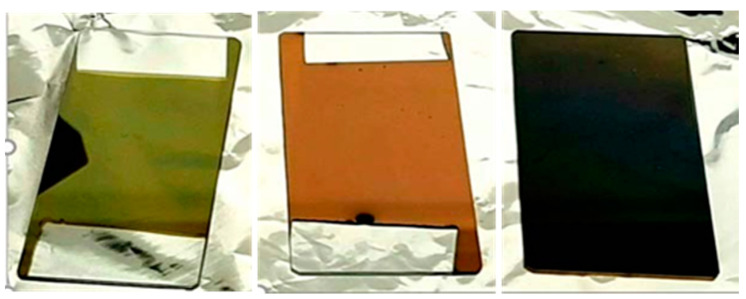
Sputtering parameters variation resulting in three different colored TiN films; green, gold, and grey (from left to right).

TiN films were prepared on four well-treated substrates; alumina (Al_2_O_3_), glass, silicon (Si), and polyimide (PI) (plastic) to investigate the adhesion behavior of TiN film. Each substrate was treated with deionized water for one hour in an ultrasonic bath prior to sputtering. Sputtering parameters, such as gas ratio, chamber pressure, and deposition power, were varied to optimize the sensor performance. Several trials of deposition runs have been conducted in various (nitrogen-deficient and nitrogen-rich) conditions and confirmed that the argon flow rate influenced the grain size, whilst the argon and nitrogen gas ratio determined the structural and electrical properties of the TiN films [29,30]. It was noted that the color of the TiN films (as shown in Figure 2) was related to the sputtering process parameters.

The pH sensing properties of green, gold, and grey colored TiN films are tabulated in Table 2. The pH sensitivity was determined by the slope of the potential difference (mV) versus time (seconds), the intercept of the slope was the E^0^ potential, hysteresis was the difference in the electrochemical potentials measured at the same pH level, and drift was the difference between the peak potential value and the 90% value of the saturated potential. The gold film exhibited Nernstian sensitivity of −59 mV/pH while, both the green and grey films had a sub-Nernstian pH sensitivity of −55 mV/pH. The gold film exhibited the best characteristics with the least hysteresis and drift of 1.2 mV and 3.9 mv/h, respectively. Consequently, the gold-colored film was adopted as the optimal material, and its color chromaticity and sensing properties were subsequently investigated.

#### Color Chromaticity Properties of As-Deposited TiN Films

The color chromaticity properties of as-deposited TiN thin films were characterized using a Konica Minolta 508D colorimeter. The chromaticity values of the gold TiN thin film were characterized based on the Hunter ***L***, ***a***, ***b*** color scale. The explanation of the Hunter Lab color space is presented in references [31,32]. The ***L*** value represents the black and white color depending on the values (0–100), and ***a*** and ***b*** values represent red to green and yellow to blue, respectively. Table 3 reports the correlation between the film color and the process parameters.

The measured Hunter Lab values of two gold TiN film samples (sputtered time was around 60 min +/− 1 min) are shown in Table 4. In this work, to analyze the color of TiN thin film, the ***L*** value was considered rather than the ***a*** and ***b*** values, as the specific film’s color will mostly be determined by the color evaluated by the human eye. The accuracy of measured ***L*** values to the simulated values was between 0.07–0.1% as indicated by the grey colored film. However, the measured ***a*** and ***b*** values for both samples were found to be positive, and that agreed with the simulated values indicating a red and yellow color, respectively, and thus confirmed the gold color of the TiN film [31].

### 3.2. Effect of TiN Film Thickness

It is well known that the film or layer thickness of the pH sensing materials has an influence on pH sensitivity [33]. We have measured the TiN film thicknesses during the deposition process using a quartz microbalance sensor, which was installed inside the sputtering chamber and reconfirmed by repetitive multiple sputtering trails. The thickness of electrodes was varied from 25 nm to 500 nm and hydrated in a pH 7 buffer for one week to eliminate any effects of aging. Electrodes were then equilibrated for 1 h at pH 7 and looped from pH 12 to 2 with pH 7 between each step in 90 s intervals. The thickness results, displayed in Table 5, show that the sensitivity remained close to Nernstian (−58.6 mV/pH at 22 °C) values for all thicknesses, except 20 nm. A precision value (defined as hysteresis to sensitivity ratio) of 0.05 was deemed acceptable for many pH applications. An 85 nm thick TiN layer provided a precision of 0.06 pH, which represents the minimum adequate thickness for most pH applications. Notwithstanding, it can also be seen from Table 5 that electrodes with thicknesses of 100 and 200 nm would be incorporated in applications that require a precision value better than 0.05. There was no consistent trend observed for electrode hysteresis with the increment of the TiN layer thickness. However, a notable increase in the drift parameter was observed when the layer thickness increased from 85 nm to 500 nm. The 85 nm thick TiN electrode was selected as the optimal since it exhibited the least amount of drift, with a Nernstian sensitivity of −59.1 mV/pH and a precision of 0.06 pH.

#### Measurement of the TiN Film Thickness

**Figure 3:**
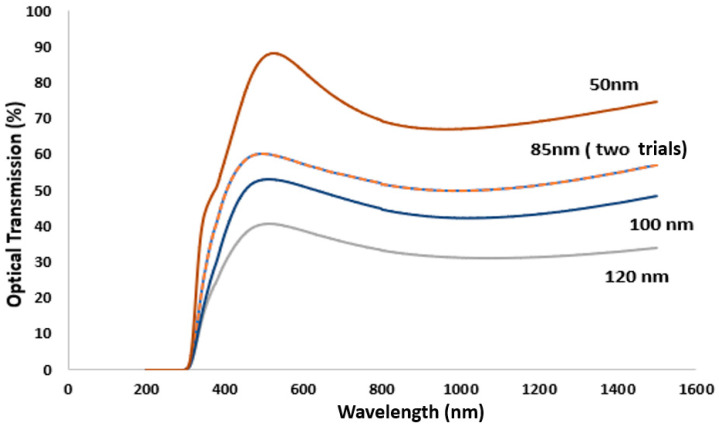
Transmission spectra of TiN films deposited onto a clear glass substrate, using 350 watts RF power to the Ti target under 2 mT pressure with the Ar (10 sccm) and N_2_ (2 sccm) gas flow.

The optical transmission spectra of as-deposited TiN films were measured using an Agilent Cary 5000 spectrometer. As discussed in Section 3.2, a thickness of 85 nm to 100 nm showed a consistent increase in drift and hysteresis as the thickness escalated. Figure 3 shows that the film thickness exhibited a transmission band in the visible region (380–750 nm), and as the thickness decreased, the transmission percentage and width of the wavelength band increased. These findings agree with those reported by Y.L. Jeyachandran et al. [34]. The 85 nm thick film was tested twice to check the thickness reading from the microbalance sensor. As shown in Figure 3, the optical spectrum of both 85 nm TiN films overlaps each other, confirming the accuracy of the film thickness readings obtained in Section 3.2.

### 3.3. Microstructure Characterization of the Sputtered TiN Film

**Figure 4:**
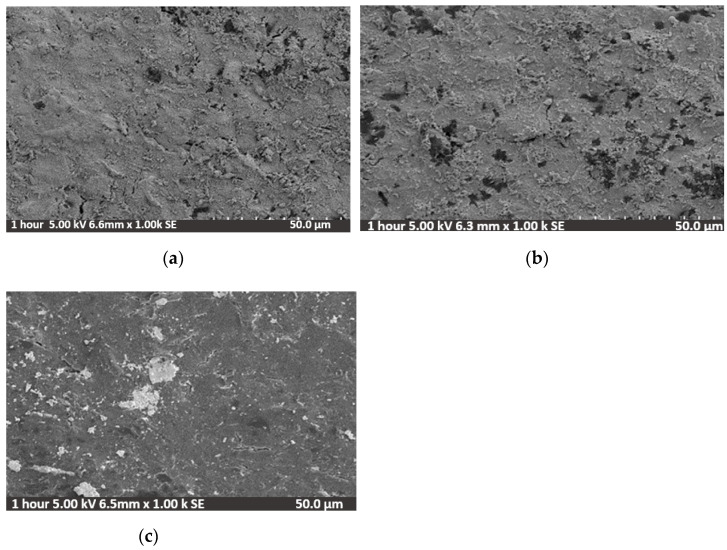
SEM images of TiN films deposited on the glass substrates using different process parameters: (**a**)**,** TiN surface magnified · 1000 prepared at 9:1 (Ar:N_2_) gas ratio, (**b**) TiN surface at 10:2 (Ar:N_2_) gas ratio and (**c**) TiN surface showing smooth morphology prepared at 10:10 (Ar:N_2_) gas ratio.

**Figure 5:**
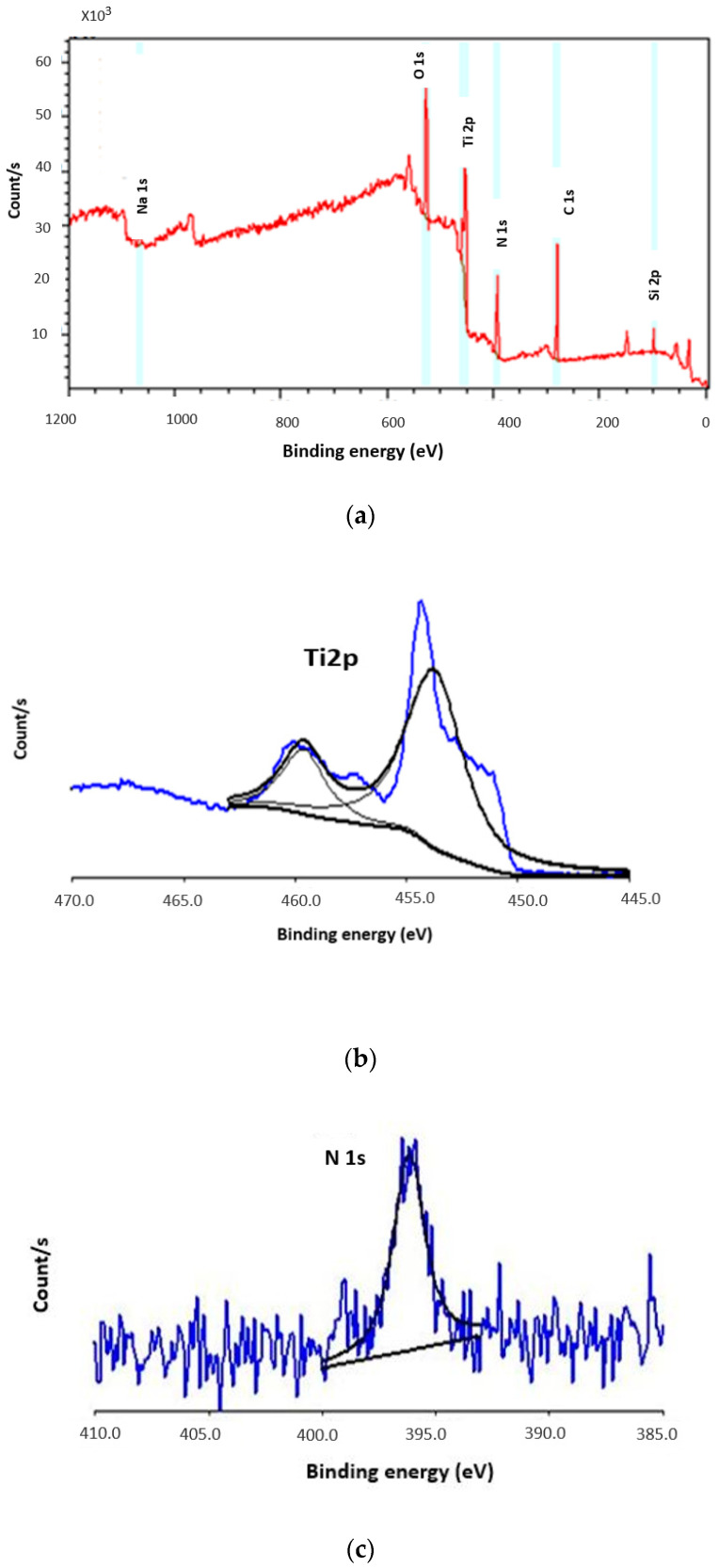

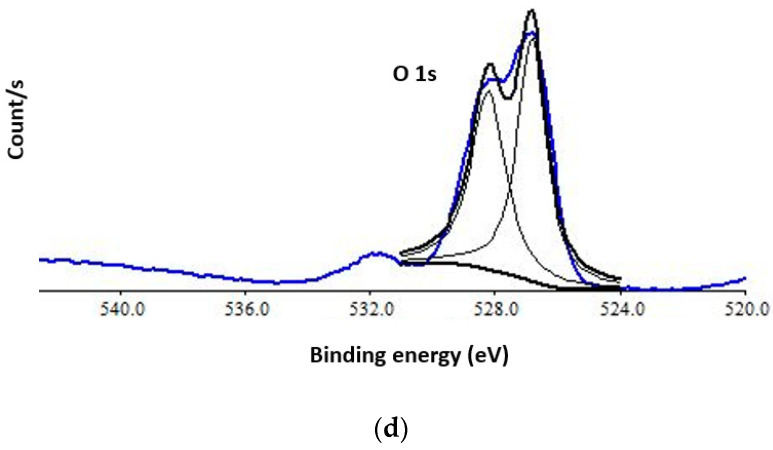
XPS characterization of 85 nm TiN film: (**a**) XPS survey spectra of the unetched 85 nm sputtered TiN film (**b**) XPS spectrum of 2p states of titanium (**c**) XPS spectrum of 1 s states of nitrogen (**d**) XPS spectrum of oxygen.

Hitachi SU3500 Scanning Electron Microscopy (SEM) was used to observe the microstructural development of TiN films on glass substrate prepared by RF magnetron sputtering. The samples were cleaned with isopropyl alcohol and dried in a hot place completely before analysis. Figure 4 shows the SEM images of the as-obtained 85 nm TiN films deposited with different Ar/N_2_ ratios resulting in green, grey, and gold films. The microstructural growth of TiN films is completely dependent on the sputtering gas ratio, and different nitrogen ratios flowing into the sputtering chamber resulted in different roughness of the surface and electrical conductivities [30]. It was found that as the nitrogen concentration was low (Ar/N_2_ = 9:1, 10:2), the surface appeared rough with crystalline TiN formations, Figure 5a,c, whereas when the nitrogen concentration was increased (Ar/N_2_ = 10:10), the surface became smoother and there are no crystalline TiN formations, Figure 5c. There was a relationship between the structural characteristics observed with the SEM and the sensing capabilities of these electrodes. The electrodes deposited with ratios Ar/N_2_ = 9:1 and 10:2 exhibited Nernstian sensitivity while the Ar/N_2_ = 10:10 sputtered electrode gave sub-Nernstian sensitivity. The gas flow inside the sputtering chamber during deposition (Ar:10, N_2_:2 sccm) resulted in a Nernstian sensitivity with low hysteresis and drift, as discussed in Section 3.1, Table 2). TiN has a special crystal structure, where the presence of interstitial atoms creates many holes within the lattice. Nitrogen atoms in the metal nitrides occupy the interstitial sites in the metal and promote stronger metal-to-non-metal and metal-to-metal bonding. In addition, hydrogen ions can diffuse between these holes, resulting in a potential difference inside and outside the crystal, as reported in Ref. [35], thus increasing the pH sensitivity of the TiN film.

### 3.4. X-ray Photoelectron Spectroscopy Analysis

The X-ray Photoelectron Spectroscopy (XPS) survey spectra of unetched 85 nm thick TiN prepared at 2 mT sputter pressure and Ar:10, N_2_:2 sccm showed that the chemical composition of the surface of the film was: Carbon, oxygen, titanium, and nitrogen (Figure 5a). The detailed characteristics of the main elements revealed Ti2p, O1s, and N1s peaks, as shown in Figure 5b–d, respectively. The C 1 s peaks seen in the spectra are most likely to be from organic carbon, Si 2p and Na 1s is from the unetched TiN surface on a glass substrate as Na atoms can easily diffuse onto the surface because of its increased mobility factor.

Figure 5b shows two peaks at binding energies of 458.9 eV and 454.5 eV. These peaks correspond to the presence of TiO_2_ (Ti^4+^) and TiN (Ti^3+^) (2p_3/2_) compounds [36,37]. The atomic percentages of Ti2p and N1s are 39.85% and 60.1%, respectively. There was no evidence of metallic titanium present on the surface since no peak was observed at 459.7 eV [38]. The N1s peak at 396.1 eV corresponds to TiN and the O1s peak at 529.1 eV belongs to TiO_2_ [39,40]. No hydroxide compounds were found in O1s spectra, and the source of oxygen seen in the spectra can be from the gas medium in the sputtering chamber or from atmospheric contamination. The surface analysis of TiN shows that titanium in the oxide form is actually thermodynamically more favorable to form than titanium nitride in this sputtering environment. This explains the presence of TiO_2_ in the titanium spectra.

### 3.5. Sensor Performance

**Figure 6:**
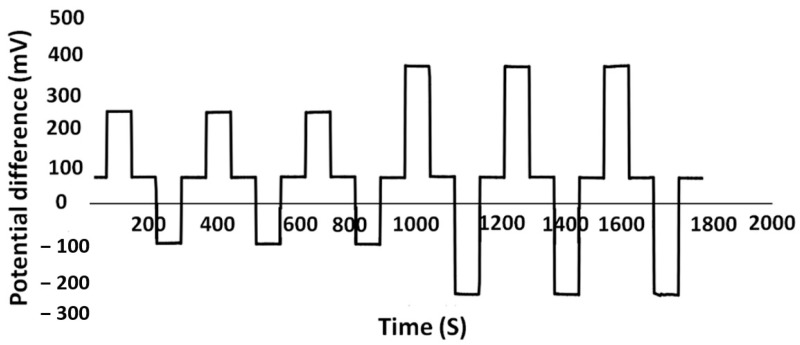
Potential recording for the developed pH sensor in pH buffers 7−2−7−12−7, and 7−4−7−10−7 three times.

**Figure 7:**
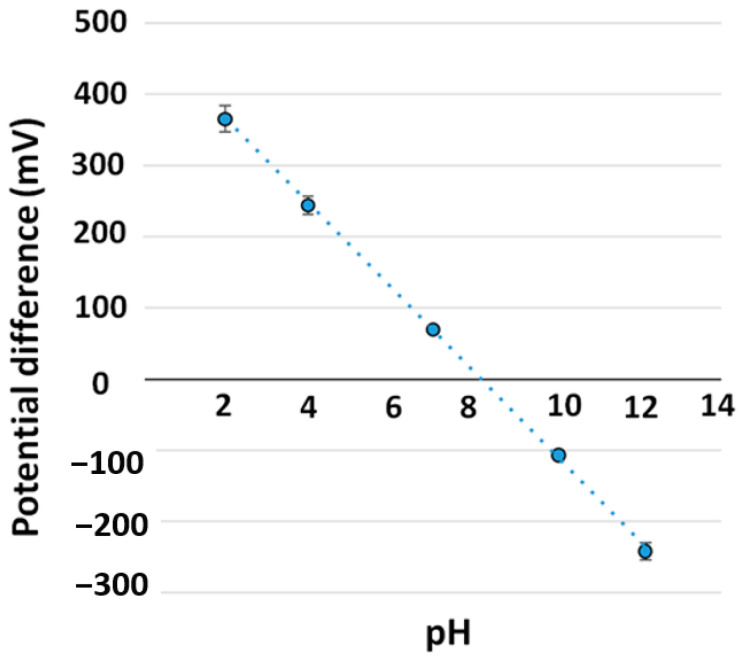
Linear calibration plot for pH sensor from pH 2 to 12, using data from Table 5.

The sensitivity of the developed TiN-based pH sensor was evaluated by immersing it in a test buffer from 2 to 12 at 22 °C. The Nernstian sensitivity of −59.1 mV/pH was obtained by incorporating a glass electrode as the reference electrode. The developed pH sensor was evaluated by looping pH from 2 to 12, as shown in Figure 6. The developed sensor shows close to the Nernstian sensitivity (−59.1 mV/pH). Table 6 and Figure 7 show the main properties and the pH response (potential versus pH value) of the developed TiN pH sensor. As seen in Figure 7, the pH response of the sensor was linear (R^2^ = 0.9997).

### 3.6. Redox Effects

**Figure 8:**
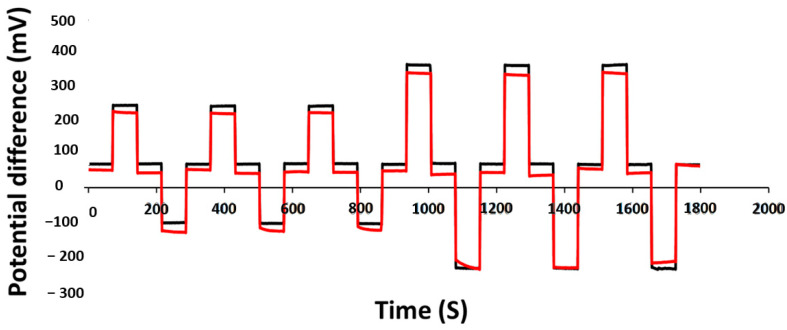
Potential versus time recording for TiN working electrode in pH 7 buffer (**black**), reducing agent (**red**) vs. glass reference electrode, pH cycled 7−4−7−10−7 and 7−2−7−12−7 three times.

The redox effects in a pH sensor involve oxidation or reduction of redox species interfering with the response of the sensor. In order to evaluate the performance of the TiN sensor in redox conditions, we compared it with a high redox sensitivity metal oxide sensor; IrO_2_. Both these sensors were left in pH 7 to equilibrate for one week, and then, the pH loops were repeated from 2 to 12 in an oxidizing agent (1 mM KMnO_4_) and reducing agent (1 mM ascorbic acid) for 20 min duration each. The sensitivity and E^0^ values of both TiN (85 nm) and IrO_2_ (100 nm) under redox conditions are recorded in Table 7. It should be noted that after oxidation, there was a decrease in sensitivity and approximately ± 450 mV increases in the E^0^ value for both TiN and IrO_2_ electrodes as noted in Table 7. After immersing the TiN electrode in a reducing solution, the sensitivity remained Nernstian with a slight decrease of ±33 mV in E^0^ value when the electrode was equilibrated at both pH 7 and pH 7 with a reducing agent for 15 min before the pH was looped from 7−4−7−10−7 and 7−2−7−12−7 three times, as illustrated in Figure 8. On the other hand, for the same conditions, the IrO_2_ electrode exhibited a sub-Nernstian sensitivity with a significant decrease of ±114 mV in E^0^ value.

**Figure 9:**
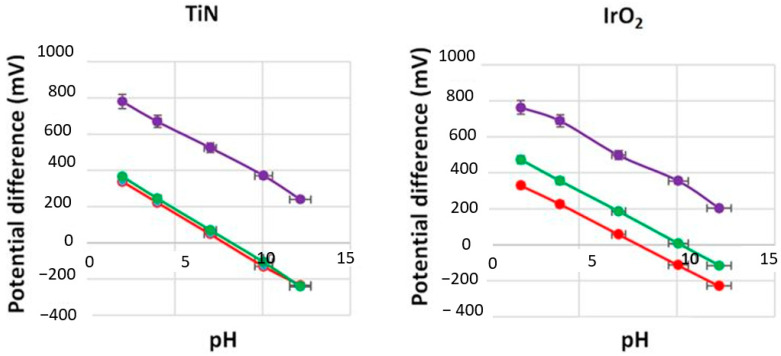
Potential versus pH value for TiN working electrode when oxidized (**purple**), pH 7 equilibrated (**green**) and reduced (**red**), linear calibration plot, when oxidized (**purple**), pH 7 equilibrated (**green**) and reduced (**red**) for RuO_2_ and IrO_2_ working electrodes.

In references [41,42], W. Lonsdale et al. have reported that for a RuO_2_ electrode, the E^0^ value can shift by approximately ±350 mV when “fully” oxidized/reduced. To address this issue, modification of RuO_2_ electrodes with a thin layer of Ta_2_O_5_ (150 nm) was shown to be effective in eliminating the interference caused by dissolved oxygen. Whilst electrode modification using a thin layer of Nafion reduced the interference from redox agents, it increased the reaction times at neutral and basic pH values [41]. The RuO_2_ electrode modified with Ta_2_O_5_|Nafion was not immune to all redox species. It was more stable in potential when compared to an unprotected electrode in matrices such as beer. Consequently, the presence of ascorbic acid in samples like white wine and fresh orange juice makes the modified RuO_2_ working electrode still inapplicable [42]. Hence, the lower potential shift of the developed TiN in reducing species, as shown in Figure 9, makes the developed TiN films more suitable for accurate pH sensing in complex matrices.

In summary, the experiments conducted with the developed TiN sensing electrode confirm that TiN based pH sensors exhibit lower redox interference and accurately obtain pH readings in reducing matrices, such as wine and fresh citrus juice, in comparison with the widely used metal-oxide pH sensors, such as the RuO_2_ [30,34,35] and IrO_2_ based pH sensors.

### 3.7. Sample Applications

Section 3.6 shows that the TiN film has a significantly lower redox shift of only ± 33 mV, as compared to metal oxides, and thus it could be applied as a potential pH sensor in strong redox samples like white wine and fresh orange juice. Hence, an unprotected 85 nm TiN working electrode was tested in redox samples with different substrates such as alumina (Al_2_O_3_), glass, silicon (Si), and polyimide (PI) (plastic). The electrode showed a 10 min reaction time initially to equilibrate until the potential difference (E^0^) was consistent. When the equilibration time was repeated a few times to check for reproducibility, unfortunately, it was noticed that the TiN layer peeled off from the glass, Si and PI substrates, but not from the 1 mm thick Al_2_O_3_. The adhesion issue will need to be considered when designing commercial metal nitride pH sensors.

**Figure 10:**
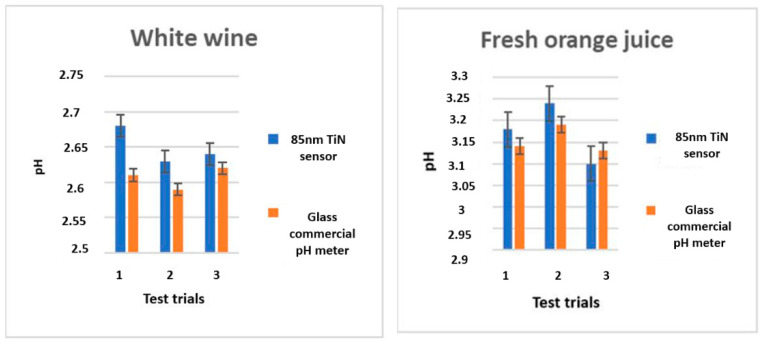
The pH values of 85 nm TiN cycled three times, and the pH values determined by a commercial pH meter in white wine and fresh orange juice matrices.

The pH values in each sample were calculated using a pH 4 buffer and with the sensitivity of −59.1 mV/pH. Figure 10 shows the pH values recorded using an 85 nm TiN unmodified electrode and a commercial glass pH sensor (EU Tech). The average pH readings of both sensors were the same within experimental error, having a precision of 0.03 pH units. In addition, the response time of TiN (10 min) was half of that of RuO_2_ and IrO_2_ (approximately 20 min) [43]. Furthermore, the equilibration duration of TiN could be reduced by modifying the TiN layer with redox blocking membranes, which will broaden the application field of this sensor where fast response time is required. These modifications not only improve the sensors reaction time but also increase the proton exchange activity in the cell, adhesion, and overall performance [44,45].

## 4. Conclusions

In this study, solid-state potentiometric TiN working electrodes have been developed and used in conjunction with a glass reference electrode to realize a pH sensor. Experimental results have shown that an 85 nm-thick TiN pH electrode exhibits a Nernstian response (−59.1 mV/pH, R^2^ = 0.9997) and excellent reproducibility (hysteresis 1.2 mV). The TiN electrode has demonstrated excellent durability with a stable Nernstian response over a 6-month period. The glass, silica, polyamide, and alumina showed similar adhesion behavior during the fabrication and characterization of TiN. Notwithstanding, in a strong redox matrix environment, alumina outperformed among the four-substrates showing the best adhesion. The colorimeter values and transmission spectrum confirmed the stochiometric thickness (85 nm) and gold color of the optimal TiN thin film electrode. SEM images showing the change in the TiN crystal structure due to different gas ratio trails have been presented, which enable the development of highly durable pH sensors with Nernstian sensitivity. Furthermore, experimental results have shown that the zero potential (E^0^) shift for the developed TiN electrode in reducing agents is only 30 mV, compared to 115 mV for IrO_2_ as their counterpart. The sample application analysis showed that TiN pH sensor is more viable for monitoring sample matrices that have reducing agents, e.g., wine and fresh citrus juice, because of its lower response time compared to metal-oxide sensors.

## Figures and Tables

**Table 1:** Summary of the process parameters and conditions used to prepare the TiN thin film layers on various substrates.

Process Parameters	Values and Comments
Sputtering target stoichiometry	Titanium Nitride (TiN, 99.95%)
Base pressure (Torr)	4–5 × 10^−6^
Argon (Ar) and Nitrogen (Ni) pressure (during deposition)	≈2 mTorr
Argon (Ar) and Nitrogen (Ni) gas flow ration	Ar:10 sccm and N_2_; 2 sccm
RF power densities	300 W
Substrate stage temperature (°C)	Room Temperature (21–23 °C)
Substrate stage rotation rate (rpm)	10–11
Substrates to target distance	15.5–16 cm

**Table 2:** pH sensing properties of colored TiN films.

Film Color	Sensitivity (mV/pH)	Hysteresis (mV)	Drift (mv/h)	E^0^ (Potential) (mV)
Green	−59	2.8	8	476
Gold	−59.1	1.2	3.9	483
Grey	−55.2	9.1	3.4	423

**Table 3:** Sputtering parameters for Titanium Nitride different colored thin film deposition.

Film Colors	Gas Ratio (Ar: N_2_) (Sccm)	Sputter Pressure (Milli Torr)	Sputter Power (Watts)
Green	09:01	10	200
Gold	10:02	2	350
Grey	10:10	10	350

**Table 4:** Measured and simulated color values of TiN films.

TiN Films	Simulated Hunter Values			Measured Hunter Values		
	*L*	*a*	*b*	*L*	*a*	*b*
Gold film 1	43.85	5.1	12.43	43.75	7.23	8.69
Gold film 2	40.67	7.8	6.07	40.60	6.68	6.96

**Table 5:** Summary of 25 to 500 nm TiN electrode in the 2–12 pH range, equilibrated at pH 7.

Thickness (nm)	Sensitivity (mV/pH)	E^0^ (mV)	R^2^	Hysteresis (mV)	Drift (mV/h)	Precision (pH)
20 nm	−49.2	498	0.9972	5.8	18	0.1
50 nm	−57.5	335.1	0.9991	5.5	9	0.1
85 nm	−59.1	483	0.9997	1.2	3.9	0.06
100 nm	−58.3	450	0.9996	1.8	4.8	0.03
200 nm	−57.5	411.3	0.9999	0.8	5.4	0.01
300 nm	−58.2	399.4	0.9998	1.3	11.8	0.2
500 nm	−58.5	442.8	0.9995	2.4	15.5	0.2

**Table 6:** Summary of the pH sensing performance of the developed TiN pH sensor. The data are extracted from Table 5.

Slope (mV/pH)	E^0^ (mV)	R^2^	Hysteresis (mV)	Drift (mV/h)	Resolution (pH)
−59.1 ± 0.1	483 ± 2	0.9997	1.2 ± 0	3.9	0.06

**Table 7:** Sensitivity(mV/pH) and E^0^ values for 85 nm TiN electrode, after exposure to pH 7 buffer, 1 mM KMnO4 (oxidized) and 1 mM ascorbic acid (reduced).

Matrix	Titanium Nitride			Iridium Oxide		
	(mV/pH)	E^0^ (mV)	R^2^	(mV/pH)	E^0^ (mV)	R^2^
pH 7	−59.1	483	0.9997	−57.9	590	0.9997
Reduced (ascorbic acid)	−56.9	451	0.9992	−55.2	444	0.9965
Oxidized (KMnO4)	−54	902	0.9974	−55	889	0.9683

## Data Availability

The data presented in this study are available on request from the corresponding author. The data are not publicly available due to further study will be carried out using the same data.
